# The Relationship Between Brain-Derived Neurotrophic Factor and Serotonin in Major Depressive and Bipolar Disorders: A Cross-Sectional Analysis

**DOI:** 10.7759/cureus.70728

**Published:** 2024-10-02

**Authors:** Mian Rohail Hayat, Muhammad Umair, Hina Ikhtiar, Shandana Wazir, Ameena Palwasha, Maheen Shah

**Affiliations:** 1 Department of Psychiatry, Mardan Medical Complex, Medical Teaching Institution (MTI), Mardan, PAK; 2 Department of Physiology, Gomal Medical College, Medical Teaching Institution (MTI), Dera Ismail Khan, PAK; 3 Department of Biochemistry, Kabir Medical College, Gandhara University, Peshawar, PAK; 4 Department of Anatomy, Bacha Khan Medical College, Mardan, PAK; 5 Department of Physiology, Jinnah Medical College, Peshawar, PAK; 6 Department of Physiology, Abbottabad International Medical Institute, Abbottabad, PAK

**Keywords:** bipolar disorder, brain-derived neurotrophic factor, brain-derived neurotrophic factor (bdnf), major depressive disorder, mood disorders, serotonin

## Abstract

Background

Mood disorders like major depressive disorder (MDD) and bipolar disorder (BD) involve complex interactions between brain-derived neurotrophic factor (BDNF) and serotonin. While extensive research has explored these factors individually, their combined effects and interactions in these disorders are less understood. This study uniquely addresses this gap by examining how BDNF and serotonin interact and relate to mood disorder severity, providing new insights into their joint role in MDD and BD.

Objectives

The objective of this study was to examine the correlation between serum BDNF and plasma serotonin levels and to assess how these correlations relate to the severity of symptoms and overall disease severity in MDD and BD.

Methodology

This cross-sectional study, conducted at the Khyber Medical University, Peshawar, from January to September 2023, examined the correlation between BDNF and serotonin in individuals with MDD and BD. Participants (n = 63) aged 18-65 were recruited based on the Diagnostic and Statistical Manual of Mental Disorders, Fifth Edition (DSM-5) criteria, excluding those with neurological disorders, substance abuse, or severe medical illness. A control group of 21 healthy individuals was matched by age and gender. Data collection involved demographic details, clinical history, and comorbid diagnoses assessed using the Mini International Neuropsychiatric Interview (MINI). Mood disorder severity was measured using the Hamilton Depression Rating Scale (HAM-D) for MDD and the Young Mania Rating Scale (YMRS) for BD, along with additional assessments (Beck Depression Inventory, Global Assessment of Functioning). Serum BDNF and serotonin levels were analyzed using enzyme-linked immunosorbent assay (ELISA) kits. Statistical analyses included t-tests, Mann-Whitney U tests, Pearson correlations, and subgroup analyses to assess relationships between biomarkers, mood disorder severity, and influencing factors.

Results

BDNF levels were found to be 20.1 ± 5.3 ng/mL in MDD, 18.5 ± 4.7 ng/mL in BD, and 25.9 ± 6.2 ng/mL in controls. Serotonin levels were 45.8 ± 12.6 ng/mL in MDD, 43.2 ± 11.4 ng/mL in BD, and 52.1 ± 14.3 ng/mL in controls. In the MDD group, significant negative correlations were observed between BDNF levels and mood disorder severity (r = -0.32, p = 0.045), whereas serotonin levels did not show significant correlations (r = -0.21, p = 0.23). In the BD group, BDNF levels also showed a significant negative correlation with manic symptoms (r = -0.28, p = 0.048), but serotonin levels showed no significant correlation. Subgroup analyses revealed that participants under 40 years had higher BDNF levels (22.8 ± 5.6 ng/mL) compared to those aged 40 and above (19.7 ± 4.3 ng/mL). Females showed higher BDNF levels (24.5 ± 6.3 ng/mL) than males (19.3 ± 3.8 ng/mL). Participants not on medication had higher BDNF levels (23.6 ± 5.1 ng/mL) compared to those on medication (17.9 ± 4.2 ng/mL). Those without comorbidities also had higher BDNF levels (23.8 ± 5.9 ng/mL) than those with comorbidities (18.2 ± 4.5 ng/mL), while serotonin levels varied similarly across these subgroups.

Conclusion

Lower BDNF levels are associated with mood disorders and symptom severity, indicating their potential as a biomarker.

## Introduction

Mood disorders involve persistent mood disturbances affecting daily life. Major depressive disorder (MDD) is characterized by prolonged sadness and loss of interest, while bipolar disorder (BD) includes alternating episodes of depression and mania [1,2]. More than 264 million people suffer from major depressive disorder (MDD), making them the third leading cause of years lived with disability [3]. The World Health Organization estimates that 45 million people worldwide have bipolar disorder [4]. The cause of mood disorders is complex and involves multiple factors, including genetics, environment, and neurobiology [4]. Brain-derived neurotrophic factor (BDNF) and serotonin have received significant attention in relation to the various neurobiological mechanisms involved [5,6]. BDNF, a neurotrophin crucial for the growth, upkeep, and adaptability of neurons, has been intensively studied in relation to mood disorders [7]. Similarly, the development of these illnesses has largely focused on serotonin, a neurotransmitter involved in mood, emotion, and cognition. While the serotonin hypothesis of depression remains controversial, several lines of evidence suggest alterations in serotonergic activity in mood disorders, particularly in depression [8].

BDNF and serotonin have a crucial bidirectional relationship in mood regulation. BDNF supports serotonin neurons by enhancing their survival and synaptic plasticity, crucial for mood stability. Conversely, serotonin regulates BDNF expression through its effects on neurogenesis and synaptic transmission. Disruptions in this interaction can contribute to mood disorders [9,10]. Serotonergic drugs, like selective serotonin reuptake inhibitors (SSRIs), increase serotonin activity, which in turn elevates BDNF levels, supporting neuronal health and improving mood [11]. This leads to increased serotonin availability at specific serotonin receptors, particularly 5-HT1A receptors. Activation of 5-HT1A receptors initiates downstream signaling pathways, such as the cAMP response element-binding protein (CREB) pathway, which ultimately upregulates the expression of BDNF. This elevation in BDNF supports synaptic plasticity, neuronal health, and mood regulation, linking SSRI use to improvements in mood disorder symptoms. Disruptions in either system can exacerbate mood disorders, with depressive symptoms often associated with lower BDNF levels and altered serotonin levels that vary between depression and BD, reflecting their different roles in these conditions [12]. Antidepressant medications, such as SSRIs, are believed to work by increasing serotonin activity, which subsequently elevates BDNF levels and improves neuroplasticity [13].

Despite several studies, the specific mechanisms by which BDNF and serotonin interact to influence mood disorders remain incompletely understood [14,15]. Prior studies have largely investigated BDNF and serotonin separately, with limited exploration of their combined effects [15,16]. Thus, there is a need for more comprehensive assessments that clarify the direct relationships between these neurobiological components and the severity of mood disorders. This study aims to address this gap by examining the interaction between BDNF and serotonin within the context of mood disorders. It will assess the levels of both substances and correlate these findings with the severity of the condition and its symptoms.

Research objective

The objective was to examine the interaction between BDNF and serotonin in mood disorders, assessing their levels and correlation with disease severity and symptoms.

## Materials and methods

The cross-sectional study was conducted at the Department of Physiology, Khyber Medical University, Peshawar, Pakistan, from January to September 2023, following ethical approval from the Institutional Review Board of Khyber Medical University (Approval No. DIR/KMU-EB/EA/000560), with informed consent obtained from all participants, and adhered to STROBE guidelines to ensure comprehensive and transparent reporting.

Participants with MDD and BD were aged 18-65 years, diagnosed according to the Diagnostic and Statistical Manual of Mental Disorders, Fifth Edition (DSM-5) criteria, and recruited from the outpatient psychiatric department. Individuals with neurological disorders, substance abuse, or severe medical illnesses were excluded. The healthy control group consisted of individuals aged 18-65 years with no psychiatric history, confirmed via a structured diagnostic interview. A total of 63 participants were included, with 21 in each group (MDD, BD, and healthy controls), matched by age and gender. The sample size was calculated based on an expected medium effect size and aimed for a power of 0.80 at an alpha level of 0.05, aligning with the prevalence rate of mood disorders in Peshawar.

Mood disorder severity was assessed using the Hamilton Depression Rating Scale (HAM-D) for depressive symptoms and the Young Mania Rating Scale (YMRS) for manic symptoms in BD patients. Both scales showed high reliability, with Cronbach’s alpha values ranging from 0.70 to 0.93. The Beck Depression Inventory (BDI) and Global Assessment of Functioning (GAF) scale were also used for comprehensive symptom and functioning assessments. Participants' demographic and clinical histories were collected via structured interviews and medical records. Mood disorder diagnoses were confirmed using the Mini International Neuropsychiatric Interview (MINI). Blood samples were collected and analyzed for BDNF and serotonin levels using enzyme-linked immunosorbent assay (ELISA) kits. 

Statistical analyses were performed using SPSS version 25 (IBM Corp., Armonk, NY). Descriptive statistics characterized demographic and clinical attributes, while independent-sample t-tests and Mann-Whitney U tests compared BDNF and serotonin levels across MDD, BD, and control groups. Pearson correlation analysis evaluated relationships between BDNF, serotonin levels, and mood disorder severity, as measured by HAM-D and YMRS scales. Subgroup analyses were also conducted to explore the influence of demographic and clinical factors on these biomarkers.

## Results

Table 1 provides a comprehensive overview of the demographic characteristics of participants across three groups: those with MDD, BD, and healthy controls, each consisting of 21 participants. The average age is reported as 42.5 ± 10.3 years for the MDD group, 38.9 ± 7.8 years for the BD group, and 40.2 ± 9.5 years for the healthy controls. Gender distribution shows that 15 (71%) of the MDD group are female, compared to 9 (43%) in the BD group and 14 (67%) in the healthy controls. Regarding education, 8 (38%) of the MDD group have a high school education, while 11 (52%) of the BD group and 7 (33%) of the healthy controls fall into this category; college education is reported for 13 (62%) of the MDD group, 10 (48%) of the BD group, and 14 (67%) of the healthy controls. Additional characteristics include duration of illness, duration of untreated illness, number of episodes, current medication status, and the presence of medical and psychiatric comorbidities, highlighting key differences and similarities among the groups.

**Table 1:** Demographic Characteristics of Participants Across Groups MDD, major depressive disorder; BD, bipolar disorder.

Characteristic		MDD Group (n = 21)	BD Group (n = 21)	Healthy Controls (n = 21)
Age (years)		42.5 ± 10.3	38.9 ± 7.8	40.2 ± 9.5
Age at Onset (years)		34.2 ± 8.1	30.5 ± 6.7	-
Gender (n, %)	Female	15 (71%)	9 (43%)	14 (67%)
	Male	6 (29%)	12 (57%)	7 (33%)
Education Level	High School	8 (38%)	11 (52%)	7 (33%)
	College	13 (62%)	10 (48%)	14 (67%)
Duration of Illness (years)		8.3 ± 4.6	10.2 ± 5.1	-
Duration of Untreated Illness (years)		2.1 ± 1.2	3.0 ± 1.5	-
Number of Episodes (Median (IQR))		4 (2-6)	5 (3-7)	-
Current Medication		18 on Antidepressants	19 on Mood Stabilizers	0
Medical Comorbidities (n, %)		7 (33%)	6 (29%)	4 (19%)
Psychiatric Comorbidities (n, %)		5 (24%)	8 (38%)	2 (10%)

The analysis of serum levels of BDNF and serotonin reveals differential correlations with mood disorder severity when measured by both the HAM-D and the YMRS (Table 2). For MDD, a significant negative correlation was observed between BDNF levels and HAM-D scores (r = -0.32, p = 0.045), indicating that lower BDNF levels are associated with greater depressive severity. However, the correlation between serotonin levels and HAM-D scores was weaker and not statistically significant (r = -0.21, p = 0.23). In BD patients, BDNF levels also showed a significant negative correlation with YMRS scores (r = -0.28, p = 0.048), suggesting that reduced BDNF is associated with more severe manic symptoms, whereas the correlation between serotonin levels and YMRS was not significant (r = -0.19, p = 0.31). This pattern suggests that BDNF may be a more robust biomarker for mood disorder severity, particularly in depressive and manic episodes.

**Table 2:** Subgroup Analyses of BDNF and Serotonin Levels BDNF, brain-derived neurotrophic factor; HAM-D, Hamilton Depression Rating Scale; YMRS, Young Mania Rating Scale.

Subgroup		BDNF Levels (Mean ± SD or Median (IQR))	Serotonin Levels (Mean ± SD or Median (IQR))	HAM-D Correlation (r, p-Value)		YMRS Correlation (r, p-Value)	
				BDNF	Serotonin	BDNF	Serotonin
Age Group	<40	22.8 ± 5.6	48.3 ± 9.2	-0.30, p = 0.055	-0.22, p = 0.20	-0.25, p = 0.07	-0.18, p = 0.15
	≥40	19.7 ± 4.3	42.7 ± 10.1				
Gender	Female	24.5 ± 6.3	55.6 ± 12.4	-0.34, p = 0.039	-0.19, p = 0.27	-0.28, p = 0.05	-0.17, p = 0.22
	Male	19.3 ± 3.8	48.9 ± 8.7				
Medication Status	On	17.9 ± 4.2	41.5 ± 10.8	-0.29, p = 0.062	-0.20, p = 0.24	0.26, p = 0.08	-0.22, p = 0.19
	Off	23.6 ± 5.1	49.2 ± 13.5				
Comorbidities	Present	18.2 ± 4.5	47.9 ± 11.3	-0.31, p = 0.048	-0.18, p = 0.33	-0.29, p = 0.052	-0.20, p = 0.29
	Absent	23.8 ± 5.9	52.3 ± 13.7				

Subgroup analyses in Table 2 further underscore the influence of demographic and clinical factors on BDNF and serotonin levels. Younger participants (<40 years) exhibited higher BDNF (22.8 ± 5.6 ng/mL) and serotonin (48.3 ± 9.2 ng/mL) levels compared to older participants (≥40 years) (BDNF: 19.7 ± 4.3 ng/mL; serotonin: 42.7 ± 10.1 ng/mL). Females had higher levels of both BDNF (24.5 ± 6.3 ng/mL) and serotonin (55.6 ± 12.4 ng/mL) compared to males (BDNF: 19.3 ± 3.8 ng/mL; serotonin: 48.9 ± 8.7 ng/mL). Additionally, participants on medication showed lower levels of BDNF (17.9 ± 4.2 ng/mL) and serotonin (41.5 ± 10.8 ng/mL) compared to those not on medication (BDNF: 23.6 ± 5.1 ng/mL; serotonin: 49.2 ± 13.5 ng/mL). The presence of comorbidities was associated with lower BDNF levels (18.2 ± 4.5 ng/mL) compared to participants without comorbidities (23.8 ± 5.9 ng/mL), although serotonin levels did not significantly differ based on comorbidity status.

Table 3 displays the correlation coefficients between serum levels of BDNF and serotonin and mood disorder severity, as measured by the HAM-D. The correlation coefficient between BDNF levels and mood disorder severity was -0.32, indicating a significant negative correlation (p = 0.045). In contrast, the correlation coefficient between serotonin levels and mood disorder severity was -0.21, which did not work to achieve statistically significant significance (p = 0.23). These results suggest a potentially stronger association between BDNF levels and mood disorder severity compared to serotonin levels.

**Table 3:** Serum Levels of BDNF and Serotonin and Their Correlation With Mood Disorder Severity BDNF, brain-derived neurotrophic factor; HAM-D, Hamilton Depression Rating Scale; MDD, major depressive disorder; BD, bipolar disorder.

Group	BDNF Levels (ng/mL)	Serotonin Levels (ng/mL)	Correlation With Mood Disorder Severity (HAM-D)	p-Value
MDD	20.1 ± 5.3	45.8 ± 12.6	BDNF: -0.32	BDNF: p = 0.045
			Serotonin: -0.21	Serotonin: p = 0.23
BD	18.5 ± 4.7	43.2 ± 11.4	-	-
Healthy Controls	25.9 ± 6.2	52.1 ± 14.3	-	-

Table 3 presents the serum levels of BDNF and serotonin across three groups: individuals with MDD, BD, and healthy controls. Serum BDNF levels are reported as mean ± standard deviation (ng/mL): 20.1 ± 5.3 for MDD, 18.5 ± 4.7 for BD, and 25.9 ± 6.2 for healthy controls. Similarly, serotonin levels are presented as mean ± standard deviation (ng/mL): 45.8 ± 12.6 for MDD, 43.2 ± 11.4 for BD, and 52.1 ± 14.3 for healthy controls.

These findings highlight that both BDNF and serotonin levels are influenced by multiple factors, with BDNF showing stronger correlations with mood disorder severity across different subgroups.

## Discussion

This study provides critical insights into the roles of BDNF and serotonin in mood disorders, specifically MDD and BD. We observed lower levels of BDNF in individuals with MDD (20.1 ± 5.3 ng/mL) and BD (18.5 ± 4.7 ng/mL) compared to healthy controls (25.9 ± 6.2 ng/mL). A significant negative correlation between BDNF levels and mood disorder severity (r = -0.32, p = 0.045) suggests that reduced BDNF levels are associated with more severe symptoms. Although serotonin levels were also lower in mood disorder groups (MDD: 45.8 ± 12.6 ng/mL, BD: 43.2 ± 11.4 ng/mL) compared to controls (52.1 ± 14.3 ng/mL), no significant correlation was found between serotonin levels and severity (r = -0.21, p = 0.23). Additionally, subgroup analyses highlighted age, gender, medication status, and comorbidities as influential factors on BDNF and serotonin levels.

The observed reduction in BDNF levels aligns with previous research indicating that decreased BDNF is a characteristic feature of mood disorders. Earlier studies have demonstrated that lower BDNF levels are linked to the pathophysiology of both depression and BD, supporting its potential as a biomarker for these conditions [14,17]. Moreover, the significant correlation between BDNF levels and mood disorder severity in our study is consistent with findings that associate reduced BDNF with more severe depressive symptoms [18]. Conversely, the lack of a statistically significant correlation between serotonin levels and mood disorder severity diverges from the serotonin hypothesis of depression, which postulates serotonin dysregulation as a central mechanism in mood disorders [19]. This discrepancy might indicate a more intricate role for serotonin, involving multiple receptors and pathways, or it could reflect the limitations of peripheral measurements of serotonin compared to central nervous system levels [20].

The lower BDNF levels observed in mood disorder groups could be attributed to BDNF's role in neuronal plasticity and survival. BDNF reduction may impair neuroplasticity, contributing to the development and maintenance of mood disorders [18]. Neuroimaging studies further corroborate this, showing structural brain abnormalities associated with low BDNF levels in mood disorders [15]. On the other hand, the lack of a significant association between serotonin levels and mood disorder severity might suggest that serotonin's role in mood disorders is more complex than previously thought. For instance, serotonin levels in peripheral blood may not accurately reflect central serotonin activity, which could be affected by numerous receptors, transporters, and environmental factors [20]. Additionally, subgroup analysis findings of age and gender differences in BDNF levels may be related to age-related neurobiological changes and hormonal influences, respectively. BDNF levels naturally decline with age, potentially increasing vulnerability to mood disorders in older individuals [21-23]. Gender differences might be influenced by hormonal fluctuations as estrogen is known to modulate BDNF expression [24].

These findings have important implications for both research and clinical practice. First, they underscore the potential of BDNF as a diagnostic biomarker for mood disorders and a target for therapeutic interventions. Given the significant association between lower BDNF levels and increased symptom severity, interventions aimed at increasing BDNF expression, such as certain antidepressants, physical exercise, and psychotherapy, may help alleviate symptoms. However, the lack of a significant correlation between serotonin levels and mood disorder severity suggests that serotonin-targeting treatments may need to consider additional pathways or mechanisms beyond simply altering serotonin levels. Future research should explore the complex roles of both BDNF and serotonin in mood disorders to develop more targeted and effective treatments.

Strengths and limitations

This study contributes uniquely to understanding the combined effects of BDNF and serotonin in mood disorders by examining their levels and correlations with disease severity. However, several limitations should be noted. The cross-sectional design limits causal inference, preventing us from definitively determining whether changes in BDNF and serotonin levels are the causes or effects of mood disorders. The relatively small sample size may limit the generalizability of the findings and the power to detect subtle differences, especially in subgroup analyses. Additionally, using convenience sampling and excluding participants with neurological disorders, substance abuse, or severe medical conditions may restrict the applicability of these results to the broader clinical population where such comorbidities are common. Potential measurement issues also exist, as serum BDNF and plasma serotonin levels can be influenced by factors like diet, physical activity, and medications, which were not controlled in this study.

Despite these limitations, the study provides valuable insights and lays the groundwork for future research. Future studies could benefit from larger, more diverse cohorts to enhance statistical power and generalizability. Longitudinal designs may help clarify causal relationships between BDNF, serotonin, and mood disorder development or progression. Additionally, controlling for potential confounders such as lifestyle factors and medication use and including a broader range of clinical populations with comorbid conditions would provide a more comprehensive understanding of the roles of BDNF and serotonin in mood disorders.

## Conclusions

This study clarifies the prominent role of BDNF in mood disorders, as the data demonstrate reduced BDNF levels in persons with MDD and BD in comparison to healthy controls. The strong inverse connection between BDNF levels and the severity of mood disorders highlights the potential of BDNF as both a diagnostic indicator and a target for therapeutic interventions. While the mood disorder groups had reduced serotonin levels, there were no significant associations found between these levels and the severity of the condition. This indicates that BDNF may serve as a more dependable biomarker. These findings support the need to create individualized therapy approaches that target BDNF modulation in order to enhance outcomes for people with mood disorders.

## Appendices

**Table 4:** STROBE Checklist of Current Study

Section	Item	Description
Title and Abstract	1	"Cross-sectional study examining BDNF and serotonin levels in MDD and BD and their association with mood disorder severity."
Introduction	2	Background: Explores interactions between BDNF and serotonin in MDD and BD; identifies research gaps in their combined effects.
Objectives	3	To examine correlation between serum BDNF and plasma serotonin levels, and their relation to symptom severity in MDD and BD.
Study Design	4	Cross-sectional study.
Setting	5	Conducted at Khyber Medical University, Peshawar, January-September 2023.
Participants	6	63 participants (21 MDD, 21 BD, 21 healthy controls), aged 18-65, DSM-5 criteria, exclusions for neurological, substance abuse, severe medical conditions.
Variables	7	Main variables: Serum BDNF and plasma serotonin levels, mood disorder severity (HAM-D for MDD, YMRS for BD).
Data Sources/Measurement	8	Serum BDNF and serotonin levels measured using ELISA; mood disorder severity using HAM-D, YMRS; demographic and clinical data collected via interviews.
Bias	9	Potential bias due to convenience sampling; controlled for by matched controls and exclusion criteria.
Study Size	10	Sample size determined via power analysis: 63 participants.
Quantitative Variables	11	BDNF and serotonin levels analyzed in relation to mood disorder severity and symptomatology.
Statistical Methods	12	Independent-samples t-tests, Mann-Whitney U tests, Pearson correlation analysis, SPSS v25.
Participants	13	63 participants recruited; inclusion/exclusion criteria applied.
Descriptive Data	14	Descriptive statistics: Mean age, gender distribution, education level.
Outcome Data	15	BDNF and serotonin levels: MDD (20.1 ± 5.3, 45.8 ± 12.6 ng/mL), BD (18.5 ± 4.7, 43.2 ± 11.4 ng/mL), Controls (25.9 ± 6.2, 52.1 ± 14.3 ng/mL).
Main Results	16	Negative correlation between BDNF and mood disorder severity (r = -0.32, p = 0.045); serotonin levels not significantly correlated.
Other Analyses	17	Subgroup analyses by age, gender, medication status, and comorbidities.
Key Results	18	Lower BDNF levels are associated with mood disorders and symptom severity.
Limitations	19	Convenience sampling, cross-sectional design limits causality; sample size limits generalizability.
Interpretation	20	BDNF could serve as a potential biomarker for mood disorder severity; serotonin’s role is less clear.
Generalizability	21	Applicable to similar clinical settings and populations, with acknowledgment of limitations.
Funding	22	Not stated.
